# Dermoscopic Features of CD8-Positive Solitary Pagetoid Reticulosis on the Left Leg

**DOI:** 10.1155/2010/850416

**Published:** 2010-07-26

**Authors:** Reiko Suzaki, Ken Kobayashi, Sumiko Ishizaki, Mariko Fujibayashi, Masaru Tanaka

**Affiliations:** ^1^Department of Dermatology, Tokyo Women's Medical University, Medical Center East, 2-1-10 Nishi-Ogu, Arakawa-Ku, Tokyo 116-8567, Japan; ^2^Department of Pathology, Tokyo Women's Medical University, Medical Center East, 2-1-10 Nishi-Ogu, Arakawa-Ku, Tokyo 116-8567, Japan

## Abstract

Solitary pagetoid reticulosis, also known as Woringer-Kolopp disease, is a rare subtype of cutaneous T-cell lymphoma. The typical clinical presentation is a solitary, localized psoriasiform or hyperkeratotic plaque or tumor located on the extremities. It primarily affects middle-aged males. Because the clinical features of pagetoid reticulosis are indistinctive, pagetoid reticulosis may progress for years before accurate diagnosis. We reported a 57-year-old Japanese woman who presented with a 1-year history of a solitary erythematous plaque on the left leg. Dermoscopic features simulated Bowen's disease showing dotted and glomerular vessels, whitish scaly areas, and a broad negative network. Dermoscopic features of pagetoid reticulosis have never been reported. We have discussed the diagnostic significance of the observed dermoscopic findings.

## 1. Case Report

**Figure 1:**
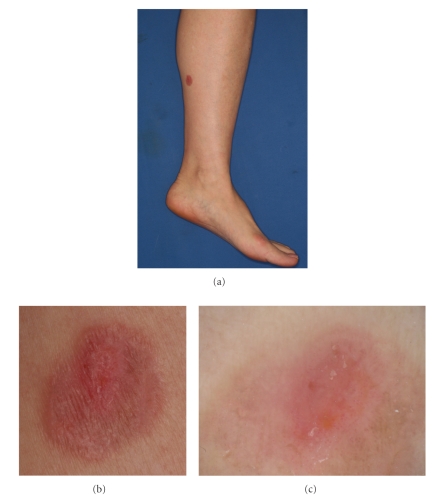
(a) A solitary slightly palpable scaly erythema on the left leg. (b) The lesion is sharply marginated with a scaly surface. (c) The dermoscopic examination demonstrated dotted and glomerular vessels with a homogenous pinkish background. A broad and indistinct whitish negative network is seen at the periphery.

**Figure 2:**
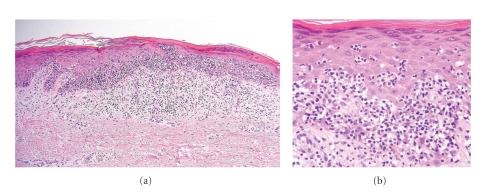
Hematoxylin and Eosin stain shows massive band-like infiltrates of atypical lymphocytes with prominent epidermotropism within a hyperplastic epidermis ((a) x100, (b) x400).

A 57-year-old Japanese woman presented with a 1-year history of a solitary erythematous plaque on the left leg (Figures 1(a) and 1(b)). She had been treated with topical corticosteroid for a year without any improvement. The physical examination revealed a solitary erythematous plaque with a scaly surface measuring 17 × 14 mm in diameter. The dermoscopic examination demonstrated dotted and glomerular vessels with a homogenous pinkish background and whitish scaly areas. At the periphery of the lesion, there is a whitish negative network simulating Bowen's disease (Figure 1(c)). However, as compared with Bowen's disease, the glomerular vessels are less prominent and the negative network is broader and more obscure. The whitish scaly areas are also less conspicuous. The histopathological examination of an excisional biopsy disclosed massive band-like infiltrates of atypical lymphocytes with prominent epidermotropism within a hyperplastic epidermis (Figures 2(a) and 2(b)). Atypical lymphocytes were positively stained with CD3 and predominantly with CD8, but negative with CD4, CD20, CD30, and CD79a. Based on histopathologic and immunohistochemical findings, a diagnosis of solitary pagetoid reticulosis was established. No evidence of metastatic lesions has been shown by brain MR imaging and chest and abdominal CT. We performed a successful operation for residual erythematous plaque with a 5 mm margin. The patient is doing well after the operation for 4 months with no recurrence or lymph node swelling.

## 2. Discussion

Solitary pagetoid reticulosis is a rare subtype of cutaneous T-cell lymphoma. It was originally described in 1939 by Woringer and Kolopp at the Clinique Dermatologique in Strasbourg, France [1]. The current WHO-EORTC classification defines pagetoid reticulosis as “a variant of mycosis fungoides characterized by the presence of localized patches or plaques with an intraepidermal proliferation of neoplastic T cells” [2]. The typical clinical presentation is a solitary, localized psoriasiform or hyperkeratotic plaque or tumor located on the extremities that demonstrates an indolent and slowly progressive clinical course. The lesional epidermis is acanthotic and infiltrated with cytologically atypical mononuclear pagetoid cells demonstrating striking epidermotropism. Immunophenotype of the neoplastic T cells is variable (CD4+/CD8−, CD4−/CD8+, CD4−/CD8−). CD30 is also often expressed [3]. In the case presented, the epidermal hyperplasia is not so prominent as observed in conventional pagetoid reticulosis and resembles more the histopathology of mycosis fungoides. For this reason, we would favor to classify this case as localized pagetoid reticulosis/unilesional (solitary) mycosis fungoides [4]. Unilesional mycosis fungoides is a solitary variant of mycosis fungoides with clinicopathologic features similar to those of “common” mycosis fungoides.

Clinically we must distinguish this disorder from other diseases, such as psoriasis vulgaris, eczema, Bowen's disease, or Paget's disease. Dermoscopy is helpful in the differential diagnosis of not only pigmented but also nonpigmented skin lesions. In this report, we have shown characteristic dermoscopic features of homogenous pinkish area in the central area of the lesion and a whitish network in the marginal area with dotted and glomerular vessels. The whitish network structure would correspond to an acanthotic epidermis. The lesion of the present case clinically and dermoscopically similated Bowen's disease but glomerular vessels were less prominent than Bowen's disease. Bowen's disease has been reported to reveal a peculiar dermoscopic pattern characterized by glomerular vessels (90%) and a scaly surface (90%) [5]. Glomerular vessels seen in dermoscopy of Bowen's disease correspond on histopathology to convolutions of grouped and frequently dilated capillaries in the papillary dermis and dermal papillae. In this case, glomerular vessels were less prominent than in Bowen's disease; in fact, on histology dilated capillaries in the papillary dermis and dermal papillae are not observed.

We have reported first experience of dermoscopic features on pagetoid reticulosis and added this disorder to differential diagnoses of Bowen's disease. Evaluations of various skin lesions using dermoscopy would be useful in the daily dermatology practice.
